# Overview of Fungi and Mycotoxin Contamination in *Capsicum* Pepper and in Its Derivatives

**DOI:** 10.3390/toxins11010027

**Published:** 2019-01-08

**Authors:** Jéssica Costa, Rodrigo Rodríguez, Esther Garcia-Cela, Angel Medina, Naresh Magan, Nelson Lima, Paola Battilani, Cledir Santos

**Affiliations:** 1 Scientific and Technological Bioresource Nucleus-BIOREN-UFRO, Universidad de La Frontera, Temuco 4811-230, Chile; j.souza01@ufromail.cl (J.C.); r.rodriguez09@ufromail.cl (R.R.); 2 Department of Sustainable Crop Production, Faculty of Agricultural, Food and Environmental Sciences, Università Cattolica del S. Cuore di Piacenza, via Emilia Parmense 84, 2910010 Piacenza, Italy; paola.battilani@unicatt.it; 3 CEB-Centre of Biological Engineering, Micoteca da Universidade do Minho (MUM), University of Minho, Campus de Gualtar, 4710-057 Braga, Portugal; nelson@ie.uminho.pt; 4 Applied Mycology Group, Environment and AgriFood Theme, Cranfield University, Cranfield, Bedford MK43 0AL, UK; m.e.garcia-cela@cranfield.ac.uk (E.G.-C.); a.medinavaya@cranfield.ac.uk (A.M.); n.magan@cranfield.ac.uk (N.M.)

**Keywords:** aflatoxins, chilli, mycotoxins, ochratoxin A, pepper, spoilage fungi

## Abstract

*Capsicum* products are widely commercialised and consumed worldwide. These substrates present unusual nutritional characteristics for microbial growth. Despite this, the presence of spoilage fungi and the co-occurrence of mycotoxins in the pepper production chain have been commonly detected. The main aim of this work was to review the critical control points, with a focus on mycotoxin contamination, during the production, storage and distribution of *Capsicum* products from a safety perspective; outlining the important role of ecophysiological factors in stimulating or inhibiting mycotoxin biosynthesis in these food commodities. Moreover, the human health risks caused by the ingestion of peppers contaminated with mycotoxins were also reviewed. Overall, *Capsicum* and its derivative-products are highly susceptible to contamination by mycotoxins. Pepper crop production and further transportation, processing and storage are crucial for production of safe food.

## 1. Introduction

Mycotoxins are low-molecular-weight secondary metabolites produced by filamentous fungi. The word mycotoxin is derived from the Greek radicals “mykes” and “toxicum”, meaning fungus and toxin, respectively. This term was coined after a veterinary outbreak in England in 1962, when approximately 100,000 turkey poultry died from the ingestion of aflatoxin-contaminated peanut meal [1]. Since then, mycotoxins have become an important issue in relation to the food safety requirements for international marketing of agri-food commodities for human and animal consumption.

Mycotoxigenic fungi grow in a wide range of agriculture crops (e.g., cereals, soybeans, grapes, tree nuts, groundnuts, coffee, cocoa and spices) and can produce one or more mycotoxins. Environmental parameters (e.g., water activity (a_w_), temperature, pH, and nutritional substrate) are the key determinants of fungal colonisation and mycotoxin biosynthesis [2].

*Capsicum* peppers are condiments widely used in cookery. As with any other agricultural crops, they are also susceptible to fungal infection and mycotoxin contamination. In the field, the phyllosphere of the growing plants are mainly colonised by yeasts and by the filamentous fungal genera *Alternaria*, *Fusarium*, *Cladosporium* and *Rhizopus*. In the harvesting and post-harvest phases, including drying, and subsequent product transportation, *Aspergillus* and *Penicillium* species are the predominant contaminants [3,4].

Mycotoxins are heat stable and difficult to remove once present in the *Capsicum* pepper production chain. Previous studies have shown that industrially processed *Capsicum* products can be contaminated with aflatoxins (AFs), ochratoxin A (OTA), fumonisins (FBs), zearalenone (ZEN), trichothecenes (TCTs), and patulin (PAT) [5]. These mycotoxins can trigger a number of acute and chronic diseases and, in more serious cases, can result in fatal consequences (e.g., most recently in Tanzania in 2016) [6].

The European Commission (EC) established the most rigorous legislation for mycotoxin in food and in feed, including regulations for AFs in *Capsicum* fruits with maximum tolerable limits (MTL) set at 10 μg/kg for total AFs (AFB_1_ + AFB_2_ + AFG_1_ + AFG_2_) and at 5.0 μg/kg for AFB_1_ [7]. The regulation was recently updated with the maximum levels of OTA in spices of 20 μg/kg for *Capsicum* powder and 15 μg/kg for mixtures of *Capsicum* with other species [8]. No maximum tolerable *Fusarium* toxin or PAT concentration has been established for pepper powder to date.

Apart from EC regulations, other countries have their own legislation, generally less comprehensive and restrictive. In Chile, the regulation for mycotoxin in spice, including *Capsicum*, establishes MTL set at 10 μg/kg, only for total AFs. Similarly, in Uruguay the legislation is established only for aflatoxins, with MTL set at 10 μg/kg for total AFs and at 5.0 μg/kg for AFB_1_. In Brazil, current legislation includes MTL established for total AFs (20 μg/kg) and OTA (30 μg/kg) in *Capsicum* spp. (dry fruit, whole or crushed, and mixtures of spices). In Pakistan, there is regulation for AFs in chilli powder with MTL fixed in 15 μg/kg for total AFs, 10 μg/kg for AFB_1_ and, 7 μg/kg for OTA. As for *Capsicum* fruits, the MTL is set at 30 μg/kg for total AFs [9]. In addition, Mexico has no legislation to control AFs and OTA in chili, a cause for concern, considering that this country is among the major exporters and consumers of *Capsicum*.

*Capsicum* peppers are commodities of economic relevance that are used in different gastronomic cultures. However, there is still a lack of information, especially on the fungus-mycotoxin-*Capsicum* pepper relationship. This review aims to summarise and discuss the current status of filamentous fungi and mycotoxins in *Capsicum* products, including the role of ecophysiological factors in mycotoxin contamination of these products. Human health risks caused by the ingestion of *Capsicum* peppers contaminated with mycotoxins will also be reviewed.

## 2. *Capsicum* Peppers

Peppers are among the most popularly consumed spices around the world. In the American continent, *Capsicum* is described as one of the oldest cultivated plants, regarded as a prehistoric crop. In the first Columbus expeditions, it was recorded that peppers were used by Mayan civilisation as a condiment and as an ingredient in medicinal preparations [10].

In Central and South America, the centre of origin and diversity of *Capsicum*, the peppers were called “*aji*” or “*chilli*” by Amerindian civilisations [11]. By the early 16th century, *Capsicum* cultivation spread throughout Europe, Asia and Africa. Due to their pungency, they were associated with black pepper, *Piper nigrum* L., although the species are not related. After its diffusion, different terms were used to refer to *Capsicum* fruits and commonly known as “red pepper”, “pepper”, “hot red pepper”, “tabasco”, “paprika”, and “cayenne” [12].

*Capsicum* is a horticultural crop produced worldwide. It belongs to one of the most valuable plant families known as Solanaceae, containing more than 3000 species that comprises many economically important plants such as tomato (*Lycopersieon esculentum* Mill.), potato (*Solatium tuberosum* L.), aubergine (*Solanum melongena*), and tobacco (*Nicotiana iobacum* L.) [10].

The *Capsicum* genus includes more than 30 species, from which *C. annuum*, *C. baccatum*, *C. chinense*, *C. frutescens* and *C. pubescens* are the most common. The plants are bushy, about 60–80 cm in height and semi-perennials, and are grown as annuals in cultivation, usually produced in tropical and sub-tropical areas [13].

*Capsicum* fruits have different shapes, colours, and degrees of spiciness, being commonly divided into two groups, pungent and non-pungent, called hot and sweet pepper. Throughout the present work, the term “*Capsicum*” will be used to refer to both spicy and non-spicy pepper; while for spicy pepper the terms red pepper or chilli will be used, and non-spicy pepper will be referred to as sweet pepper.

### Pepper Production Process

Red pepper is the second largest consumed spice throughout the world, after black pepper. Its exotic characteristics of taste, aroma, colour and pungency, as well as the multivariate forms of consumption, have made this spice widely used in gastronomy.

According to the Food and Agriculture Organization (FAO) [14], in 2016, the worldwide production area for dried *Capsicum* was 1,798,847 ha, with a production of 3,918,159 tonnes of harvested product per year. China is an important supplier of crushed and ground peppers. In 2016, China produced the highest amount of peppers, followed by Mexico and Turkey.

The production indices show the importance of *Capsicum* crop mainly in developing economies. However, the fact that they are frequently contaminated with spoilage fungi, has put at risk the income of small and large-scale pepper producers. Peppers are amongst the spices that are most susceptible to fungal contamination, especially by potentially mycotoxigenic species. Most of these commodities reach the market without undergoing proper processing. In general, they are only dried and then ground before being packaged and made available to the consumer [15,16].

**Figure 1:**
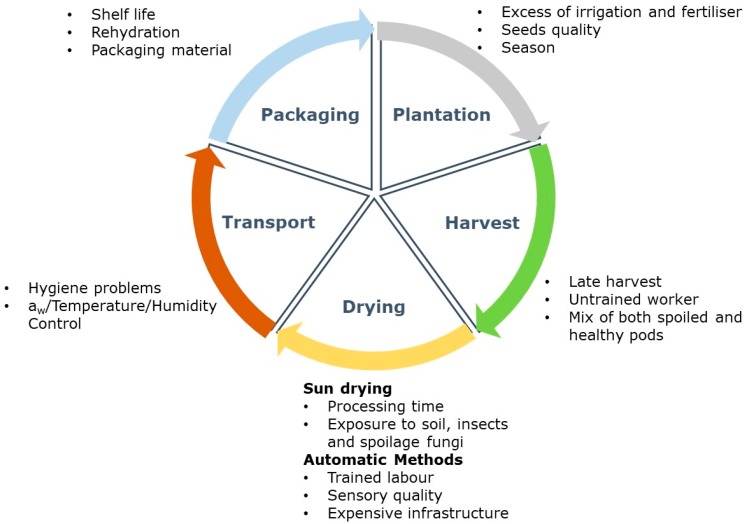
Critical factors favouring fungal growth and mycotoxin contamination during the pepper production chain.

The whole pepper production chain should be managed carefully to prevent fungal infection and mycotoxin contamination. In Figure 1, the critical factors favouring fungal growth and mycotoxin contamination during the pepper production chain are highlighted. In the sowing stage, excessive irrigation and fertiliser application above the recommended levels enhance plant susceptibility to fungal colonisation [17]. The selection criteria for pepper seeds must be rigorous; this is not always the case as the production is mainly by small rural farmers.

Harvesting of *Capsicum* fruits takes place at different stages during maturation. For the agro-industry sector, they are usually harvested fully mature [18]. At this stage of advanced maturity, the pod is susceptible to mechanical damage and to the action of phytophagous insects, sometimes vectors of fungal spores. In addition, it is recommended that red pepper moisture content (65–80% = 0.995 a_w_) at post-harvesting should be immediately reduced to around 13% (=0.50 a_w_) by drying to inhibiting enzyme activities, fungal growth, and mycotoxin contamination [19,20].

The drying and storage phases are critical in the pepper production chain. Traditionally, peppers are dried by direct sun exposure or mechanical heat drying; these methods involve extended periods of time at oscillating temperatures and humidity or at higher controlled temperatures for short time periods, respectively (Figure 1) [21].

Due to its low operational cost, pepper drying under the sun remains the most widespread method used in Asia, Africa and Central/South America. After being placed on the floor and turned several times to obtain an even drying, the pepper pods are usually smoked [18].

In China and Nigeria, artisanal drying of pepper can take between 7 and 28 days. In Chile, this process is done following traditional methods. After sun drying, peppers are placed in a traditional structure made with light wood (similar to bamboo) locally called “pidil”. Pepper is then smoked inside traditional houses, called “ruca”, which are made of wood and straw [18]. In Mexico, *Capsicum annuum* var. aviculare (locally called “chiltepín”), a wild pepper, is exposed to sun drying from 5 to 7 days [22]. Moreover, in other countries, depending on the weather, sun drying takes between 14 and 21 days. Overall, during the drying period, pods are affected by changes in temperature, exposure to dust, and wind and insect infestation. This timeframe allows fungal colonisation to occur, especially by xerophilic/xerotolerant fungi (*Aspergillus* and *Penicillium* species). In addition, poor post-harvest hygiene in storehouses can lead to further fungal colonisation and an increase in the risk of mycotoxin contamination [23].

Regarding mechanical drying, water can be removed through hot air exposure (e.g., wind chambers), sublimation (by freeze-drying method) or by means of water molecule friction caused by a magnetic field (e.g., microwave drying). Despite high energy costs, mechanical dehydration is used at an industrial scale; the temperature, air velocity and humidity controlled conditions reduces the time for microbial contamination and improves pepper quality (Figure 1) [24,25].

Packaging is the final stage of pepper production (Figure 1). For pepper samples, polyethylene bags and vacuum-packaging methods have proven to be more efficient for inhibiting water reintroduction and decreasing aeration, both factors that are important for limiting fungal growth and mycotoxin production [26,27,28].

## 3. Filamentous Fungi in *Capsicum* Pepper

Several studies have emphasised that spices are poor substrates for fungal growth [15,29]. The low a_w_ and the naturally occurring antifungal substances have been suggested to be the main barriers to microbiological development. However, high frequencies of fungi in pepper powder and other seasonings produced with *Capsicum* have been described in recent years [30,31,32,33].

The plant defence mechanisms that involve the production of antifungal substances and the low a_w_ found in processed products are key hurdles, but they do not completely prevent fungal growth. The fungal metabolic plasticity allows adaptation to adverse conditions along the *Capsicum* production chain, as shown in Table 1. This allows fungal growth in both the fresh *Capsicum* pod, where a high a_w_ level prevails, especially where natural sun drying is used, and in pepper powder, a substrate rich in NaCl, and other mixed condiments. In addition, the mycotoxigenic fungi, which can occupy the niche of intermediate moisture products, would be at an advantage in such ecological niches [34]. Other studies have found high levels of fungal contamination in peppers mainly by species belonging to *Alternaria*, *Aspergillus*, *Fusarium* and *Penicillium* (Table 1). Mandeel [3] showed that among 17 evaluated spices, red pepper was the most heavily fungal contaminated, mainly by *Aspergillus flavus* (96 strains) and *A. niger* (62 strains).

Soil-born fungi such as *Mucor*, *Cladosporium*, *Harzia* and *Rhizopus* genera have also been isolated from *Capsicum* products, but less frequently (Table 1). The presence of spoilage fungi can affect the organoleptic properties of *Capsicum* derived-products, reducing their commercial value and efficiency in culinary seasoning [35]. Besides this, a high contamination with fungi can be associated with mycotoxin contamination, which could influence consumer health. The contaminant mycotoxins are heat stable during cooking and are thus very difficult to remove once present [36].

In the field cultivation stage, soil, wind and the other growing plants are the main microbiological sources [3]. Pepper plants may have a symbiotic root-mycorrhizal relationship (e.g., *Glomus* sp. and *Gigaspora* sp.). In stressed conditions, this association promotes water and nutrients uptake [37]. However, these cultivars are also susceptible to soil-borne pathogens such as *Phytophthora capsici*, *Colletotrichum capsici* and *Botrytis cinerea*, which can cause root rot, foliar blight and pod rot and impact on the final yield [38,39].

*Alternaria* and *Fusarium* are also considered field pathogens. Species of both genera can cause rot and wilt in living plants and can infect fresh fruits, especially after injury by insects or chilling, mechanical damage, sunburn, or calcium deficiency [40,41]. The rotting of pepper fruit by these fungi can also occur in mature peppers either before or after harvest (Table 1).

After harvesting, *Capsicum* pods can be consumed fresh. However, most of the production is directed towards the processing of dried spices, often for making seasonings (e.g., sauce pepper, spice powder mix, and so forth). In order to avoid fungal infection and mycotoxin contamination in the post-harvest stage, the pepper should be dried to around 13% m.c. (=0.50 a_w_) [19,42]. Adebanjo and Shopeju [43] showed that in fresh *C. annuum*, *Fusarium equiseti* was the main species found, while in stored samples the incidence of both *A. alternata* and *Fusarium* spp. was low.

In the drying, storage, packaging and transportation stages the control of hygiene conditions, temperature, humidity and a_w_ are critical factors to guarantee a low bioburden. Since most of the fungi isolated are probably contaminants rather than originating from the native plant [44].

In these steps, *Aspergillus* and *Penicillium* species are the main spoilage fungi. These species are found in a high frequency of isolation from *Capsicum* by-products at the marketing stage (e.g., factory production, restaurants, supermarkets, retailers markets). Besides this, these species may also be present in latent forms in the pepper plant. However, due to their xerophilic characteristics, in low a_w_ conditions they acquire a competitive advantage in relation to other fungal pathogens [20,45].

Small- and medium-scale pepper cultivation systems often do not have the level of control of the pepper production process, especially in relation to good handling and storage practices. Despite the low levels of moisture content (11.0% to 16.3%) and a_w_ (0.513 to 0.611), Casquete et al. [46] isolated 67 fungal species from smoked paprika samples. Both potential aflatoxigenic *Aspergillus* species and PAT-producer *Penicillium* (*P. expansum* and *P. thomii*) were found. In these samples, mycotoxins were not detected, agreeing with other studies that had already stated that a_w_ conditions required for the production of mycotoxins are slightly more restricted than for growth [34]. The long periods of drying and poor sanitary conditions where pepper products are stored, can contribute to this gradual increase in fungal contamination levels [46].

Similarly, red pepper samples, even after drying, can have an increase in populations of spoilage fungi with *A. glaucus*, *A. niger* and *A. fumigatus* being dominant [27,43,47]. Furthermore, in pepper producing countries such as Korea, India, Peru, Nigeria, Pakistan and Sri Lanka, climatic conditions such as high temperature, humidity, and rainfall contributed to the high fungal population loads in pepper samples [26,32,48,49].

Pepper products are very hygroscopic. Thus, after drying they need to be effectively packaged to prevent any increase in a_w_, which would allow mycotoxigenic fungi to become active and produce mycotoxins. Where pepper products are sold in markets in bulk, rehydration can occur impacting on the risk for spoilage and increased toxin contamination. In order to minimise the risk of mycotoxin contamination at the post-processing stage, effective packaging systems, including modified atmosphere systems, must be used to prevent the readsorption of moisture.

Mandeel [3] evaluated the spoilage fungal profile in 17 raw spice samples obtained from retail outlets. The author recovered and identified, from dried and ground spice samples, a total of 665 fungal strains. Samples collected from exposed gunny bags yielded the highest fungal population counts when compared to samples collected from wooden boxes, plastic bags and metal containers.

Abou-Arab et al. [50] reported that the highest percentage of *Penicillium* spp. isolation was recorded in packed spice samples, highlighting the importance of using appropriate packaging methods to decrease fungal colonisation and mycotoxin contamination.

It should be noted that although some of the aforementioned fungi have been described as mycotoxigenic, their isolation on *Capsicum* does not imply these products will necessarily be contaminated with mycotoxins. Pepper plant varieties may have different susceptibility levels to fungal colonisation and mycotoxin contamination [27].

Indeed, mycotoxin biosynthesis will be affected by the strain virulence and relative tolerance to environmental and nutritional conditions [51,52,53]. Gherbawy et al. [51] evaluated the mycobiota in *C. annuum* derivative-products, including chilli sauce, crushed chilli and chilli powder obtained from retail markets and food restaurants of Taif City, Saudi Arabia. In their studies, the authors isolated some *A. flavus* strains; while these strains had the gene clusters for aflatoxin biosynthesis, they did not appear to produce any toxins.

## 4. Mycotoxins in *Capsicum*

Pepper has extensively been reported to be frequently contaminated by mycotoxins in different countries around the world (Table 2). In Europe, in 2017 and 2018 alone, 41 cases of pepper contamination were reported by the Rapid Alert System for Food and Feed (RASFF). Of the 41 notifications concerning mycotoxins in *Capsicum*, 30 of them were classified as rejections at the border, 5 as alerts and 6 of them as information notifications. According to the RASFF [64], 33 notifications referred to AFs, and 8 to OTA.

Concerning classes of mycotoxins found in pepper and pepper derivatives, AFs and OTA are among the most important contaminants from a consumer point of view. In addition, other mycotoxins such as citrinin (CIT), deoxynivalenol (DON), FB_2_, PAT, sterigmatocystin (ST) and ZEN, and their co-occurrence, are important issues that need to be examined in more detail. They may need to be controlled in the future in the production chain, processing and consumption of pepper and pepper-based derivatives (Table 2).

### 4.1. Aflatoxins

Aflatoxins are potent carcinogenic, mutagenic and immuno-suppressive agents [65]. They are naturally occurring fungal metabolites, found in a range of food commodities originating from food/feed products, especially those originating from tropical/sub-tropical regions, as described in Table 2. To date, nearly 20 different types of AFs have been described, among which, the most frequent are AFB_1_, AFB_2_, AFG_1_ and AFG_2_. AFB_1_ is the most toxic compound, and classified as a Class 1 carcinogen.

Singh and Cotty [32] evaluated the prevalence of AFB_1_ in chillies from markets across the United States of America (USA) and Nigeria. These authors compared AFs levels in chillies from both countries and found that samples purchased in Nigeria were more contaminated than those purchased in the USA. AFB_1_ was detected in 64% chillies from USA markets (*n* = 169), and 93% of Nigerian chillies (*n* = 55).

Only 2% of the USA samples exceeded the regulatory limit of 20 µg/kg for total AFs; while the highest concentration detected was 94.9 µg/kg. AFB_1_ concentrations were significantly higher in Nigerian pepper, with the most contaminated sample containing 156 µg/kg AFB_1_. About 38% of USA chillies were contaminated with >5 µg/kg AFB_1_ (mean = 11.1 µg/kg), and based on European Union (EU) regulatory limits, all of these peppers would be rejected for importation into the EU.

Despite their excellent sensorial characteristics, Pakistani peppers have lost space in the international market because of mycotoxin contamination [17]. Moreover, previous studies have already reported higher AF levels than those established by the EU [30].

Regarding processed pepper products (e.g., crushed pepper, powdered pepper and paprika) they are more susceptible to AF contamination than the fresh fruit [27,30,51]. Reddy et al. [66] evaluated the contamination of chilli pods by AFB_1_ collected from the principal market yards and cold storage facilities of the major chilli-growing areas of Andhra Pradesh (AP, India), and in chilli powders collected from different supermarkets in Hyderabad, AP. Authors found that 59% of chilli samples collected were contaminated with AFB_1_. In contrast, the highest level of AFB_1_ contamination was found in pepper pods (969 μg/kg). This may be because pepper pods contained >40% of discoloured pods, those with insect damage and visible fungal growth. Moreover, according to further analysis, these samples were contaminated by *A. flavus*. Because of its low price, low-income people largely consume these kinds of chilli in India. Of course, high-quality powdered pepper is directly linked to a selection of high-quality pods.

AFs can be produced during any step of the pepper production chain process (Table 2). In the field, pepper pods are more susceptible to AFs contamination during the summer [67]. Moreover, even when properly stored, in the later stages (cold storage) it is still possible to detect traces of mycotoxins in pepper samples [27,68]. Özkan et al. [69] reported that in crushed pepper samples the levels of total AFs and AFB_1_ varied according to the season of feedstock collection.

### 4.2. Ochratoxin A

Following AFs, OTA is the most prominent mycotoxin found in pepper samples. Due to its immunotoxic action, nephrotoxic and carcinogenic potential, OTA contamination is of global concern. Overall, OTA is the most common mycotoxin found in food and feed. OTA has previously been reported in red pepper samples [49,70], dried chilli pod [48,71,72], chilli powder [31,73,74] red pepper flakes [75], chilli sauce [27], sweet pepper [33,76], and paprika [28,70,77,78] (see Table 2).

Geographical, climatic characteristics and crop management systems directly affect mycotoxin contamination levels. Almela et al. [26] evaluated the occurrence of OTA in peppers grown for paprika in Peru, Brazil, Zimbabwe and Spain. A total of 115 fungal strains were isolated. Of these, 85 fungal strains belonging to *Aspergillus* Section Circumdati (*A. ochraceus*) and Section Nigri (*A. niger*, *A. carbonarius*) from OTA contaminated paprika samples. Among the latter ones, about 31% (26 isolates) of the *A. ochraceus* and 1.7% (1 isolate) of the *A. niger* strains were OTA producers in vitro. According to the authors, great differences in OTA content in paprika samples were found. Moreover, a relationship with the climatic conditions of the geographic origin of the samples, and cultural and technical practices in pepper manipulation were observed. For instance, the highest OTA contamination in red pepper originated from Peru. This could be related to the high humidity conditions prevailing in the pepper growing areas.

### 4.3. Other Mycotoxins and Their Co-Occurrence

As already discussed above, different studies have reported the occurrence of other mycotoxins in pepper, such as CIT, DON, FB2, PAT, sterigmatocystin (ST) and ZEN, mainly produced by species belonging to *Aspergillus*, *Fusarium* and *Penicillium* [5,61,70]. Although mycotoxins of *Alternaria* have been less frequently reported, Cabral et al. [39] detected tenuazonic acid (TeA), alternariol (AOH) and its monomethyl (AME) in sweet pepper samples (see Table 2).

Co-occurrence of mycotoxins in *Capsicum* derived-products, such as paprika and chilli samples have previously been reported [77,78,79]. Ozbey and Kabak [74] evaluated the co-occurrence of AFs and OTA in commercial spices in Turkey. The co-occurrence of both mycotoxins was detected in 62.5% of red chilli flake, 40.9% of red chilli powder and 4.3% of black pepper powder samples. It is important to point out that, red chilli flakes and red chilli powder were found to contain the highest levels of OTA (53.04 and 98.24 µg/kg, respectively), and were simultaneously contaminated with AFB_1_ with the highest concentrations (11.45 and 35.77 μg/kg, respectively).

Santos et al. [70] evaluated the co-occurrence of AFs, OTA and ZEN in paprika and chilli samples commercialised in Spain. According to the authors, the occurrence of mycotoxins in 64 paprika was positive for AFs (59%), OTA (98%) and ZEN (39%); whereas in 35 chilli samples, the AFs, OTA and ZEN contaminations were positive for 40%, 100% and 46% of the samples, respectively. Moreover, none of the samples had AFs levels higher than the EU legislative maximum allowable limits. Regarding the co-occurrence of mycotoxins, 75% of paprika samples and 65% of chilli samples contained more than one mycotoxin.

Mycotoxin co-occurrence were evaluated for 30 ground red pepper samples obtained from 15 manufacturers in the main chilli-pepper-producing areas of South Korea [49]. OTA was detected in 3 samples (1–2 µg/kg); whereas no AFs were detected in ground red pepper samples.

Co-occurrences with multi-mycotoxins from dry chilli samples collected from the markets in Sri Lanka (*n* = 86) and Belgium (*n* = 35) has also been reported [48]. In this study, 17 chemically divergent mycotoxins were analysed. Sixty-seven per cent of the Sri Lankan samples exceeded the EU maximum level for AFB_1_ and 44% of the samples exceeded the EU limit for total AFs. Nine of the 11 positive chilli samples from Belgium exceeded the EU limit for AFB_1_. Moreover, about 33% of the Sri Lankan chillies were contaminated with more than 3 different mycotoxins. According to authors, co-occurrence of different mycotoxins [AFB_1_ and OTA (36%), AFB_1_ and ST (28%), OTA and AFB_1_ and ST (17%), and AFB_1_ and FB_2_ (14%)] was found in different forms of chilli.

ST is a known intermediate product of AFB_1_ biosynthesis; both mycotoxins are mainly produced by *Aspergillus versicolor* and *A. flavus*. Moreover, higher frequency of mycotoxin co-occurrence found in processed chillies such as flakes and powder could be due to the fraudulent usage of low-quality grade chilli pods for spice processing.

Gambacorta et al. [33] analysed the presence of 17 chemically divergent compounds in 45 sweet pepper samples collected in southern Italy. Results showed that 86% of the samples contained a number of mycotoxins ranging from 5 to 16, with a mean of 7 mycotoxins per sample. Mycotoxin co-occurrence is of concern for consumers. Combined intake of different types of mycotoxins may lead to a synergistic or at least additive effect. Currently, there is no legislation for combined contamination of mycotoxins in different foods, except for the control of the four AFs together.

## 5. Ecophysiological Modulators of Mycotoxin Biosynthesis in Pepper

*Capsicum* is a substrate with particular characteristics. In this food matrix the fungus–host interaction as well as the inhibition or increase in mycotoxin biosynthesis is markedly affected by interacting factors of a_w_, temperature, and nutritional status of food matrix [88]. There is significant evidence that a_w_ alone and interaction with temperature affect mycotoxin regulation, with some evidence that this is a response to such environmental stresses [2]. Moreover, during *Capsicum* pod storage, temperature effect can also greatly depend on the fungal species involved. Low temperatures can inhibit germination and mycotoxin production by *Aspergillus* spp. On the other hand, *Penicillium* spp. are prone to grow in low temperatures, so cold storage does not prevent the spoilage, but just delays fungal growth [68,95].

Capsaicinoids are important secondary metabolites responsible for the pepper fruit pungency and antimicrobial properties [96,97,98]. Although *Capsicum* plants produce chemical defences, spoilage and mycotoxigenic fungal strains have already been reported in several studies [98,99,100,101,102]. Very few studies have examined the ecological interactions between *Capsicum* fruits and fungal pathogens, and none has considered the role that mycotoxins might play in this interaction. Thus, it seems reasonable to suggest that the capsaicinoids group can act both in activating and/or inhibiting mycotoxin biosynthesis, while for fungi, mycotoxins may have a key role in their host–pathogen relationship, providing protection against environmental stress. Moreover, spices processed from pepper are among the foods containing higher sodium concentrations [103], which can be toxic to some spoilage and mycotoxigenic fungi [104]. Although the nutritional profile of pepper does not impede fungal growth, the high concentration of sodium in pepper powder may impose a challenge for other microbial species.

Overall, there are still unanswered questions about the *Capsicum*-fungus-mycotoxin relationship: (i) Do mycotoxins confer some adaptive strategy for the fungi in the presence of capsaicinoids? (ii) What would the role of mycotoxins be in non-pungent varieties of *Capsicum* (e.g., sweet pepper)? Therefore, would this be a more challenging environment for fungal growth in this substrate? (iii) Do the compounds from this non-pungent substrate have some antifungal activity? (iv) Does NaCl act as an external signal to trigger the mycotoxin biosynthesis in this kind of substrate? (v) Does *Aspergillus* stimulate the production of OTA by NaCl in this kind of substrate? (vi) What is the role of simultaneous action of NaCl and capsaicinoids compounds such as capsaicin in the production of OTA?

## 6. Impact of *Capsicum* spp. Mycotoxins’ Contamination on Health

*Capsicum* products are used as seasoning and consumed in reduced portions. Consequently, there is no major food outbreak related to this spice. However, experimental research has already shown that pepper consumption may be a strong risk factor for gastric cancer. López-Carrill et al. [105] suggested that capsaicin, a compound present in pepper, behaves as a carcinogen. On the other hand, the daily intake of mycotoxin-contaminated pepper is subjected to the accumulation of the toxic metabolite in the body, possible leading to acute intoxication and being translated in different kind of mycotoxin-related diseases.

Serra et al. [106] suggested that mycobiota present in red pepper may also be a risk factor for gallbladder cancer (GBC). Ikoma et al. [81] reported the contamination of red peppers obtained from Bolivia, Chile, and Peru by AFs and OTA. According to the authors, all of the peppers from the three countries showed contamination with AFs below the EC recommended limits (5 μg/kg), but the OTA contamination of two samples was above the EC recommended limit (15 μg/kg). The mean concentrations of OTA in the peppers from Chile (mean 355 μg/kg, range <5–1059 μg/kg) and Bolivia (mean 207 μg/kg, range 0.8–628 μg/kg), that have high incidence of GBC, were higher than that in Peru (14 μg/kg, range <5–47 μg/kg), which has an intermediate GBC incidence.

Similarly, Nogueira et al. [107] evaluated the plasma related to GBC cases in Chile. Authors found AFB_1_-adducts in 64% of the evaluated human blood samples (23 samples). In the study, the GBC cases were associated with the consumption of red pepper contaminated with AFB_1_. The results are not yet conclusive, but they point out the ingestion of mycotoxin-contaminated pepper as one of the determining factor for the development of GBC among the inhabitants of this country.

A prolonged exposure to mycotoxin-contaminated red peppers may trigger different acute and chronic diseases. Specific medical research such as those developed by López-Carrill et al. [105] and Serra et al. [106] is needed, especially in communities with high consumption of this spice, such as the Andean, Mexican American, and Asian populations.

## 7. Concluding Remarks and Perspectives

Despite its economic relevance, Capsicum and its derivative-products are highly susceptible to contamination by mycotoxins. AFs, OTA, ZEN, FMs and PAT, as well as less expressive mycotoxins such as DON, AME and AOH that have been detected in paprika, chilli sauce, and seasonings made out of *Capsicum*.

Pepper crop production and further transportation, processing and storage are crucial for the production of safe food. These have proven to be critical steps for the food safety of this food commodity. In these last stages, the control of a_w_, temperature and moisture content are essential to avoid the growth of potential mycotoxigenic spoilage fungi, such as *Aspergillus* and *Penicillium* species.

The presence of capsaicinoids in *Capsicum* plant and in powdered pepper can select and delay fungal infection. However, further research is needed to elucidate the ecophysiological conditions that favour fungal growth in this substrate, as well as the role that mycotoxins play during the infection process. Moreover, studying the effect of substrate composition (e.g., NaCl and capsaicinoids) on the mycotoxin production can open new avenues in knowledge of how potential mycotoxigenic fungi can be controlled in terms of their metabolism.

## 8. Materials and Methods

The literature review was performed based on analysis of scientific data published about filamentous fungi and their mycotoxins that impact on the quality of *Capsicum* pepper and in its derivatives. The fragmented information was compiled and tabulated allowing a gap analysis and identification what the problems need to be investigated in this field. The concept map for the current review started with the *Capsicum* pepper production chain and its critical factors that favouring fungal growth and mycotoxin contamination. The aflatoxins and ochratoxin A were the major mycotoxin strings used. However, other mycotoxins were also taken into consideration and tabulated. All scientific literature available were taken into consideration with special attention on publications of the last decade (2008–2018) which also represented more than 50% of the references used. Finally, the relevant databases used for worldwide production area and for mycotoxin contamination of *Capsicum* pepper were Food and Agriculture Organization of the United Nations (FAOSTAT) [14] and RASFF [64], respectively.

## Figures and Tables

**Table 1:** Fungi isolated on *Capsicum* derivative-products.

Pepper Product	Fungi Species	References
Fresh Fruit (chilli, red pepper, pepper fruits and cayenne pepper)	*Aspergillus candidus*; *A. flavus*; *A. fumigatus*; *A. glaucus*; *A. niger*; *A. ochraceus*; *A. sydowii*; *A. tamarii*; *A. terreus*; *Aspergillus* sp.; *Alternaria alternata*; *A. arborescens*; *A. armoraciae*; *A. tenuissima*; *Cladosporium herbarum*; *Cladosporium* sp.; *Epicoccum* sp.; *Fonnellia flavipes*; *Fusarium dimerum*; *Fusarium* sp.; *Geotrichum* sp.; *Harzia* sp.; *Mucor* sp.; *Penicillium chrysogenum*; *Penicillium* sp.; *Rhizopus stolonifer*; *Trichoderma hamatum*	[3,39,43,54,55]
Smoked paprika	*Aspergillus niger*; *A. oryzae*; *A. sydowii*; *Alternaria* sp.; *Fusarium graminearum*; *F. verticillioides*; *Mucor* sp.; *Penicillium citrinum*; *P. expansum*; *P. griseofulvum*; *P. raistrickii*; *P. thomii*; *Rhizopus* sp.	[5,46,56]
Dry pepper (pepperoni pepper powder, Kashmiri chili hot, Kashmiri chili mild, ground red pepper, chilli powder and paprika)	*Aspergillus niger*; *A. oryzae*; *A. sydowii*; *Alternaria* sp.; *Fusarium graminearum*; *F. verticillioides*; *Mucor* sp.; *Penicillium citrinum*; *P. expansum*; *P. griseofulvum*; *P. raistrickii*; *Rhizopus* sp.; *Botrytis cinerea*; *Chaetomium globosum*; *Fusarium moniliforme*; *F. oxysporum*; *Geotrichum candidum*; *Mucor racemosus*; *Mucor* sp.; *Mycosphaerella tassiana*; *Penicillium carneum*; *P. charlesii*; *P. chrysogenum*; *P. citrinum*; *P. crustosum*; *P. griseofulvum*; *P. verruculosum*; *P. corylophilum*; *Rhizopus arrhizus*; *R. oryzae*; *R. stolonifer*; *Scopulariopsis brevicaulis*; *Syncephalastrum racemosum*; *Syncephalastrum* sp.; *Trichoderma hamatum*; *Ulocladium chartarum*; *Wallemia sebi*	[5,15,35,49,51,57,58,59,60,61,62,63]
Chilli sauce	*Aspergillus amstelodami*; *A. flavus*; *A. candidus*; *A. chevalieri*; *A. niger*; *A. ochraceus*; *Eurotium amstelodami*; *Fusarium oxysporum*; *Mucor racemosus*; *Penicillium chrysogenum.*	[51]

**Table 2:** Mycotoxins incidence on different pepper derivative-products around the globe.

Pepper Products	Mycotoxin	Country	References
Pepper fruits/Whole chilli/Pepper pod	AFs ^a^; AOH ^c^; AME ^d^; CIT ^e^; FB ^g^; OTA ^i^; ST ^k^; TeA ^l^	Argentina; Belgium; Chile; India; Nigeria; Pakistan; Sri Lanka; Turkey	[27,32,39,48,68,69,80,81]
Red pepper flakes	AFs; CIT; FB; OTA; ST	Belgium; Turkey	[48,75]
Chilli oil	AFs; OTA; ZEN ^p^	United Kingdom	[82]
Paprika	AFs; OTA; ZEN	Australia; Brazil; Morocco; Peru; Portugal; Spain	[26,70,77,83,84,85]
Chilli/Ground chilli/Kashmiri chilli/Dried chilli	AFs; CIT; FB; OTA; ST; ZEN	Australia; Bolivia; Chile; India; Pakistan; Qatar; Saudi Arabia; Thailand Turkey; United States	[27,31,32,51,60,61,70,73,83,84,85,86,87,88]
Red pepper pastes	PAT ^j^	Turkey	[89]
Red pepper powder/Ground red pepper/*Capsicum* powder	AFs; OTA; ST	Egypt; Ethiopia; Iran; Korea; Saudi Arabia; Turkey	[28,49,57,62,90,91,92,93]
Sweet peppers pod/Bell pepper pod/Fresh sweet peppers	AFs; ALT ^b^; AME; AOH; FB; OTA; TeA ^l^; TTX ^m^; ZEN	China; India; Italy; Turkey	[33,76,94]
Dried sweet peppers	AFs; AME; AOH; DON ^f^; FB; HT-2 ^o^; NIV ^h^; OTA; TeA; TTX; ZEN	Italy	[33]
Ground sweet pepper	AFs; ALT; AME; AOH; DON; FB; H-2 ^n^; HT-2; NIV; OTA; TeA; TTX; ZEN	Italy	[33]
Fried sweet pepper	AFs; AOH; AME; DON; FB; H-2; HT-2; NIV; OTA; TeA; TTX; ZEN	Italy	[33]

^a^ Aflatoxins = AFs; ^b^ Altenuene = ALT; ^c^ Alternariol = AOH; ^d^ Alternariol monomethyl ether = AME; ^e^ Citrinin = CIT; ^f^ Deoxynivalenol = DON; ^g^ Fumonisin = FB; ^h^ Nivalenol = NIV; ^I^ Ochratoxin = OTA; ^j^ Patulin = PAT; ^k^ Sterigmatocystin = ST; ^l^ Tenuazonic acid = TeA; ^m^ Tetrodotoxina = TTX; ^n^ Trichothecene H-2 toxin; ^o^ Trichothecene HT-2 toxin; ^p^ Zearalenone = ZEN.
